# [(*Z*)-*N*-Isopropyl-*O*-methyl­thio­carbamato-κ*S*](tri-*p*-tolyl­phosphine-κ*P*)gold(I)

**DOI:** 10.1107/S1600536809046893

**Published:** 2009-11-11

**Authors:** Primjira P. Tadbuppa, Edward R. T. Tiekink

**Affiliations:** a Department of Chemistry, National University of Singapore, Singapore 117543; b Department of Chemistry, University of Malaya, 50603 Kuala Lumpur, Malaysia

## Abstract

In the title compound, [Au(C_5_H_10_NOS)(C_21_H_21_P)], two independent mol­ecules comprise the asymmetric unit, and these are connected by an aurophilic inter­action [Au⋯Au = 3.1351 (3) Å]. Each Au^I^ atom is linearly coordinated within a *S*,*P*-donor set with the distortion from ideal linear geometry [S—Au—P = 175.31 (5) and 176.45 (5)°] ascribed to an intra­molecular Au⋯O contact in each case [2.974 (4) and 3.027 (4) Å].

## Related literature

For structural systematics and luminescence properties of phosphinegold(I) carbonimidothio­ates, see: Ho *et al.* (2006 ▶); Ho & Tiekink (2007 ▶); Kuan *et al.* (2008 ▶). For the synthesis, see Hall *et al.* (1993 ▶). For related structures, see: Bott *et al.* (2004 ▶); Cookson & Tiekink (1994 ▶).
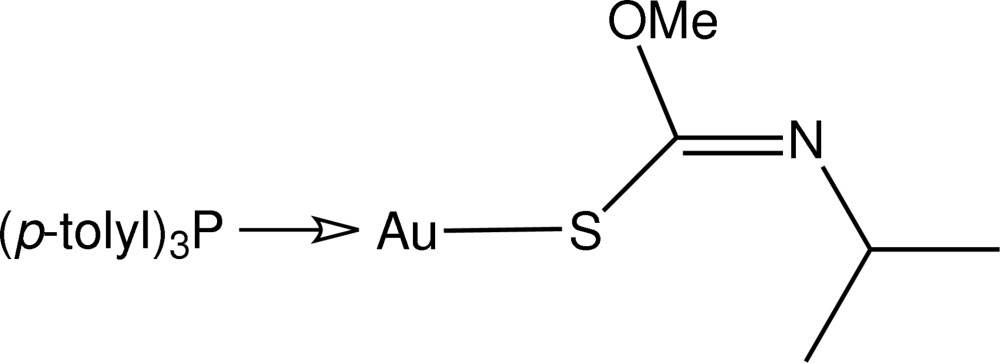


## Experimental

### 

#### Crystal data

[Au(C_5_H_10_NOS)(C_21_H_21_P)]
*M* *_r_* = 633.51
Triclinic, 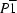

*a* = 9.6445 (4) Å
*b* = 12.7202 (5) Å
*c* = 22.995 (1) Å
α = 103.731 (1)°
β = 96.950 (1)°
γ = 98.443 (1)°
*V* = 2674.81 (19) Å^3^
*Z* = 4
Mo *K*α radiation
μ = 5.66 mm^−1^
*T* = 223 K
0.32 × 0.07 × 0.07 mm

#### Data collection

Bruker SMART CCD diffractometer
Absorption correction: multi-scan (*SADABS*; Bruker, 2000 ▶) *T*_min_ = 0.510, *T*_max_ = 1
22571 measured reflections
12259 independent reflections
9152 reflections with *I* > 2σ(*I*)
*R*_int_ = 0.033

#### Refinement

*R*[*F* ^2^ > 2σ(*F* ^2^)] = 0.040
*wR*(*F* ^2^) = 0.089
*S* = 0.97
12259 reflections
567 parameters
H-atom parameters constrained
Δρ_max_ = 1.41 e Å^−3^
Δρ_min_ = −0.52 e Å^−3^

### 

Data collection: *SMART* (Bruker, 2000 ▶); cell refinement: *SAINT* (Bruker, 2000 ▶); data reduction: *SAINT*; program(s) used to solve structure: *PATTY* in *DIRDIF92* (Beurskens *et al.*, 1992 ▶); program(s) used to refine structure: *SHELXL97* (Sheldrick, 2008 ▶); molecular graphics: *DIAMOND* (Brandenburg, 2006 ▶); software used to prepare material for publication: *SHELXL97*.

## Supplementary Material

Crystal structure: contains datablocks global, I. DOI: 10.1107/S1600536809046893/hg2584sup1.cif

Structure factors: contains datablocks I. DOI: 10.1107/S1600536809046893/hg2584Isup2.hkl

Additional supplementary materials:  crystallographic information; 3D view; checkCIF report

## Figures and Tables

**Table 1:** Selected bond lengths (Å)

Au1—S1	2.3221 (13)
Au1—P1	2.2638 (13)
Au2—S2	2.3102 (14)
Au2—P2	2.2589 (14)

**Fig. 1.**
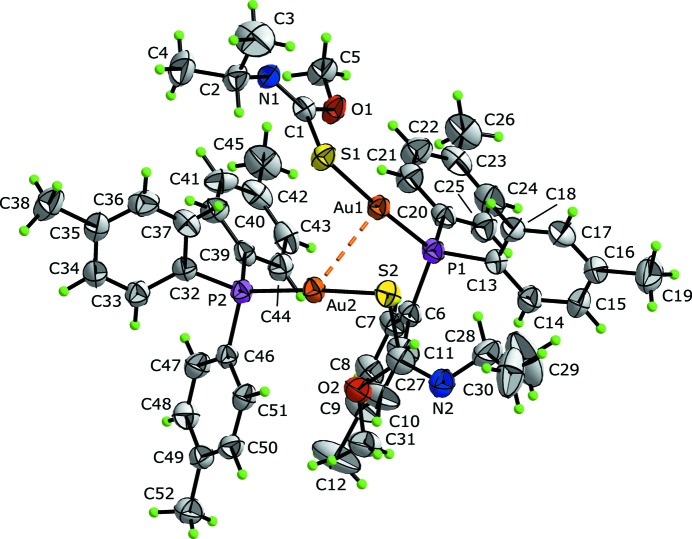
Molecular structure of (I) showing atom-labelling scheme and displacement ellipsoids at the 50% probability level.

## supplementary crystallographic information

### Comment

The structure of the title compound, (I), was determined as a part of an on-going study of the structural systematics, including luminescence properties, of molecules related to the general formula *R*_3_PAu[SC(OR') NR''] for *R*, *R*' and *R*'' = alkyl and aryl (Ho *et al.* 2006; Ho & Tiekink, 2007; Kuan *et al.*, 2008).

Two essentially equivalent molecules comprise the asymmetric unit, Fig. 1. These are connected by an aurophilic interaction of 3.1351 (3) Å. Each Au atom exists within a SP donor set and comparable geometric parameters are similar, Table 1. Deviations from the ideal linear geometry [S—Au—P = 175.31 (5) and 176.45 (5) °] are likely due to the proximity to Au of the respective O atom [2.974 (4) and 3.027 (4) Å].

As a general comment, aurophilic interactions are comparatively rare in phosphinegold(I) carbonimidothioates so the presence of a Au···Au contact in (I) prompted an examination of the structures of the precursor (*p*-tol)_3_PAuCl structures. There are two polymorphs reported for this compound for which atomic coordinates are available. In the monoclinic polymorph, no aurophilic interaction was noted (Cookson & Tiekink, 1994) but, in the orthorhombic form, Au···Au contacts [3.375 (1) Å] were observed (Bott *et al.*, 2004). Such vagaries in supramolecular aggregation underscore the difficulties in crystal engineering with these systems.

### Experimental

Compound (I) was prepared following the standard literature procedure from the reaction of (*p*-tol)_3_PAuCl and MeOC(S)N(H)-^i^Pr in the presence of base (Hall *et al.*, 1993).

### Refinement

The H atoms were geometrically placed (C—H = 0.94–0.99 Å) and refined as riding with *U*_iso_(H) = 1.2–1.5*U*_eq_(C). The maximum and minimum residual electron density peaks of 1.41 and 0.52 e Å^-3^, respectively, were located 0.96 Å and 1.25 Å from the Au2 and H29*c* atoms, respectively.

### Crystal data

**Table:**

[Au(C_5_H_10_NOS)(C_21_H_21_P)]	*Z* = 4
*M**_r_* = 633.51	*F*(000) = 1248
Triclinic, *P*1	*D*_x_ = 1.573 Mg m^−^^3^
Hall symbol: -P 1	Mo *K*α radiation, λ = 0.71069 Å
*a* = 9.6445 (4) Å	Cell parameters from 4932 reflections
*b* = 12.7202 (5) Å	θ = 2.2–24.5°
*c* = 22.995 (1) Å	µ = 5.66 mm^−^^1^
α = 103.731 (1)°	*T* = 223 K
β = 96.950 (1)°	Prism, colourless
γ = 98.443 (1)°	0.32 × 0.07 × 0.07 mm
*V* = 2674.81 (19) Å^3^	

### Data collection

**Table:**

Bruker SMART CCD diffractometer	12259 independent reflections
Radiation source: fine-focus sealed tube	9152 reflections with *I* > 2σ(*I*)
graphite	*R*_int_ = 0.033
ω scans	θ_max_ = 27.5°, θ_min_ = 0.9°
Absorption correction: multi-scan (*SADABS*; Bruker, 2000)	*h* = −11→12
*T*_min_ = 0.510, *T*_max_ = 1	*k* = −16→15
22571 measured reflections	*l* = −29→29

### Refinement

**Table:**

Refinement on *F*^2^	Primary atom site location: structure-invariant direct methods
Least-squares matrix: full	Secondary atom site location: difference Fourier map
*R*[*F*^2^ > 2σ(*F*^2^)] = 0.040	Hydrogen site location: inferred from neighbouring sites
*wR*(*F*^2^) = 0.089	H-atom parameters constrained
*S* = 0.97	*w* = 1/[σ^2^(*F*_o_^2^) + (0.0393*P*)^2^] where *P* = (*F*_o_^2^ + 2*F*_c_^2^)/3
12259 reflections	(Δ/σ)_max_ = 0.002
567 parameters	Δρ_max_ = 1.41 e Å^−^^3^
0 restraints	Δρ_min_ = −0.52 e Å^−^^3^

### Special details

**Table:**

Geometry. All s.u.'s (except the s.u. in the dihedral angle between two l.s. planes) are estimated using the full covariance matrix. The cell s.u.'s are taken into account individually in the estimation of s.u.'s in distances, angles and torsion angles; correlations between s.u.'s in cell parameters are only used when they are defined by crystal symmetry. An approximate (isotropic) treatment of cell s.u.'s is used for estimating s.u.'s involving l.s. planes.
Refinement. Refinement of *F*^2^ against ALL reflections. The weighted *R*-factor *wR* and goodness of fit *S* are based on *F*^2^, conventional *R*-factors *R* are based on *F*, with *F* set to zero for negative *F*^2^. The threshold expression of *F*^2^ > 2σ(*F*^2^) is used only for calculating *R*-factors(gt) *etc*. and is not relevant to the choice of reflections for refinement. *R*-factors based on *F*^2^ are statistically about twice as large as those based on *F*, and *R*- factors based on ALL data will be even larger.

### Fractional atomic coordinates and isotropic or equivalent isotropic displacement parameters (Å^2^)

**Table:**

	*x*	*y*	*z*	*U*_iso_*/*U*_eq_	
Au1	0.05819 (2)	1.018576 (17)	0.202900 (9)	0.03982 (7)	
Au2	0.21442 (2)	0.883806 (17)	0.279145 (9)	0.04019 (7)	
S1	0.23483 (14)	1.15335 (11)	0.26808 (6)	0.0463 (3)	
S2	0.31241 (16)	0.83442 (12)	0.19202 (7)	0.0493 (3)	
P1	−0.12358 (14)	0.89835 (12)	0.13825 (6)	0.0407 (3)	
P2	0.11746 (14)	0.92117 (11)	0.36470 (6)	0.0383 (3)	
O1	0.0144 (4)	1.2461 (3)	0.25565 (16)	0.0494 (10)	
O2	0.2647 (4)	0.6531 (3)	0.22738 (18)	0.0590 (11)	
N1	0.1861 (5)	1.3508 (4)	0.33280 (19)	0.0441 (11)	
N2	0.2885 (5)	0.6198 (4)	0.1281 (2)	0.0575 (13)	
C1	0.1452 (5)	1.2637 (4)	0.2907 (2)	0.0410 (12)	
C2	0.3239 (6)	1.3657 (5)	0.3710 (3)	0.0517 (15)	
H2	0.3515	1.2929	0.3679	0.062*	
C3	0.4325 (7)	1.4360 (7)	0.3492 (4)	0.097 (3)	
H3A	0.4384	1.4012	0.3075	0.146*	
H3B	0.5242	1.4453	0.3743	0.146*	
H3C	0.4059	1.5074	0.3517	0.146*	
C4	0.3097 (8)	1.4154 (6)	0.4356 (3)	0.087 (2)	
H4A	0.2397	1.3663	0.4484	0.130*	
H4B	0.2796	1.4857	0.4388	0.130*	
H4C	0.4006	1.4261	0.4615	0.130*	
C5	−0.0674 (6)	1.3310 (5)	0.2694 (3)	0.0559 (16)	
H5A	−0.0852	1.3411	0.3108	0.084*	
H5B	−0.1571	1.3104	0.2418	0.084*	
H5C	−0.0154	1.3991	0.2649	0.084*	
C6	−0.1889 (5)	0.7811 (4)	0.1660 (2)	0.0424 (13)	
C7	−0.3173 (6)	0.7698 (5)	0.1863 (2)	0.0461 (13)	
H7	−0.3789	0.8192	0.1819	0.055*	
C8	−0.3563 (6)	0.6857 (5)	0.2135 (3)	0.0576 (16)	
H8	−0.4450	0.6780	0.2266	0.069*	
C9	−0.2674 (6)	0.6141 (6)	0.2214 (3)	0.0631 (18)	
C10	−0.1386 (6)	0.6261 (5)	0.2006 (3)	0.0676 (19)	
H10	−0.0773	0.5765	0.2051	0.081*	
C11	−0.0982 (6)	0.7083 (5)	0.1738 (3)	0.0516 (15)	
H11	−0.0095	0.7156	0.1607	0.062*	
C12	−0.3091 (8)	0.5257 (7)	0.2527 (4)	0.102 (3)	
H12A	−0.3863	0.5431	0.2745	0.153*	
H12B	−0.2282	0.5216	0.2811	0.153*	
H12C	−0.3396	0.4554	0.2228	0.153*	
C13	−0.0834 (5)	0.8408 (4)	0.0641 (2)	0.0414 (12)	
C14	−0.1605 (6)	0.7446 (5)	0.0245 (2)	0.0515 (15)	
H14	−0.2332	0.7026	0.0374	0.062*	
C15	−0.1322 (7)	0.7094 (5)	−0.0336 (3)	0.0587 (17)	
H15	−0.1869	0.6443	−0.0597	0.070*	
C16	−0.0253 (7)	0.7681 (5)	−0.0540 (3)	0.0569 (16)	
C17	0.0555 (7)	0.8621 (5)	−0.0140 (3)	0.0666 (18)	
H17	0.1316	0.9016	−0.0264	0.080*	
C18	0.0258 (7)	0.8989 (5)	0.0442 (3)	0.0547 (15)	
H18	0.0804	0.9640	0.0703	0.066*	
C19	0.0082 (9)	0.7287 (7)	−0.1170 (3)	0.090 (3)	
H19A	0.0455	0.6611	−0.1203	0.135*	
H19B	0.0784	0.7844	−0.1248	0.135*	
H19C	−0.0776	0.7153	−0.1464	0.135*	
C20	−0.2751 (5)	0.9646 (5)	0.1259 (2)	0.0436 (13)	
C21	−0.2828 (6)	1.0623 (5)	0.1661 (3)	0.0550 (15)	
H21	−0.2093	1.0943	0.1991	0.066*	
C22	−0.3984 (7)	1.1136 (5)	0.1580 (3)	0.0629 (17)	
H22	−0.4012	1.1800	0.1861	0.076*	
C23	−0.5084 (6)	1.0716 (6)	0.1108 (3)	0.0542 (15)	
C24	−0.5006 (7)	0.9719 (6)	0.0714 (3)	0.0667 (19)	
H24	−0.5753	0.9394	0.0389	0.080*	
C25	−0.3873 (6)	0.9194 (5)	0.0783 (3)	0.0593 (16)	
H25	−0.3857	0.8523	0.0506	0.071*	
C26	−0.6321 (7)	1.1279 (6)	0.1024 (3)	0.078 (2)	
H26A	−0.6949	1.1158	0.1311	0.116*	
H26B	−0.6835	1.0983	0.0614	0.116*	
H26C	−0.5983	1.2063	0.1094	0.116*	
C27	0.2870 (5)	0.6894 (5)	0.1768 (3)	0.0477 (14)	
C28	0.3114 (10)	0.6566 (6)	0.0745 (3)	0.083 (2)	
H28	0.3403	0.7377	0.0855	0.100*	
C29	0.1795 (11)	0.6229 (10)	0.0303 (4)	0.141 (4)	
H29A	0.1055	0.6576	0.0475	0.212*	
H29B	0.1508	0.5435	0.0205	0.212*	
H29C	0.1952	0.6451	−0.0062	0.212*	
C30	0.4262 (9)	0.6030 (8)	0.0468 (3)	0.111 (3)	
H30A	0.5142	0.6257	0.0754	0.167*	
H30B	0.4400	0.6254	0.0100	0.167*	
H30C	0.3982	0.5236	0.0370	0.167*	
C31	0.2423 (8)	0.5370 (5)	0.2195 (3)	0.0708 (19)	
H31A	0.1562	0.5028	0.1906	0.106*	
H31B	0.2328	0.5207	0.2581	0.106*	
H31C	0.3225	0.5083	0.2043	0.106*	
C32	0.2406 (5)	0.9995 (4)	0.4321 (2)	0.0394 (12)	
C33	0.2445 (6)	0.9743 (5)	0.4878 (2)	0.0475 (13)	
H33	0.1847	0.9112	0.4910	0.057*	
C34	0.3342 (6)	1.0403 (5)	0.5380 (3)	0.0504 (14)	
H34	0.3363	1.0205	0.5749	0.060*	
C35	0.4221 (6)	1.1353 (5)	0.5361 (2)	0.0499 (14)	
C36	0.4205 (6)	1.1581 (5)	0.4805 (3)	0.0604 (17)	
H36	0.4813	1.2207	0.4774	0.072*	
C37	0.3328 (6)	1.0924 (5)	0.4297 (3)	0.0531 (15)	
H37	0.3350	1.1104	0.3924	0.064*	
C38	0.5130 (7)	1.2097 (6)	0.5927 (3)	0.074 (2)	
H38A	0.4636	1.2678	0.6099	0.111*	
H38B	0.6018	1.2419	0.5828	0.111*	
H38C	0.5324	1.1676	0.6218	0.111*	
C39	−0.0258 (5)	0.9982 (4)	0.3607 (2)	0.0401 (12)	
C40	−0.0188 (6)	1.1040 (5)	0.3965 (3)	0.0515 (14)	
H40	0.0615	1.1376	0.4261	0.062*	
C41	−0.1287 (6)	1.1601 (5)	0.3889 (3)	0.0572 (16)	
H41	−0.1212	1.2322	0.4130	0.069*	
C42	−0.2499 (6)	1.1126 (6)	0.3465 (3)	0.0542 (15)	
C43	−0.2567 (6)	1.0071 (6)	0.3119 (3)	0.0572 (16)	
H43	−0.3379	0.9730	0.2829	0.069*	
C44	−0.1479 (6)	0.9502 (5)	0.3188 (2)	0.0500 (14)	
H44	−0.1564	0.8779	0.2948	0.060*	
C45	−0.3702 (7)	1.1759 (6)	0.3397 (3)	0.077 (2)	
H45A	−0.4153	1.1848	0.3757	0.115*	
H45B	−0.4396	1.1356	0.3044	0.115*	
H45C	−0.3326	1.2477	0.3349	0.115*	
C46	0.0408 (5)	0.7956 (4)	0.3820 (2)	0.0386 (12)	
C47	−0.0665 (6)	0.7922 (5)	0.4171 (2)	0.0504 (14)	
H47	−0.1031	0.8561	0.4318	0.060*	
C48	−0.1200 (6)	0.6952 (5)	0.4307 (3)	0.0518 (14)	
H48	−0.1953	0.6936	0.4531	0.062*	
C49	−0.0646 (6)	0.6010 (4)	0.4121 (2)	0.0460 (13)	
C50	0.0425 (6)	0.6054 (4)	0.3774 (2)	0.0476 (14)	
H50	0.0813	0.5420	0.3641	0.057*	
C51	0.0944 (6)	0.7007 (4)	0.3618 (2)	0.0446 (13)	
H51	0.1660	0.7009	0.3375	0.054*	
C52	−0.1245 (7)	0.4963 (5)	0.4267 (3)	0.0653 (17)	
H52A	−0.2129	0.4620	0.3995	0.098*	
H52B	−0.1423	0.5127	0.4683	0.098*	
H52C	−0.0571	0.4465	0.4219	0.098*	

### Atomic displacement parameters (Å^2^)

**Table:**

	*U*^11^	*U*^22^	*U*^33^	*U*^12^	*U*^13^	*U*^23^
Au1	0.03911 (12)	0.04092 (12)	0.03790 (12)	0.00767 (9)	0.00326 (9)	0.00853 (9)
Au2	0.03970 (12)	0.04677 (13)	0.03748 (12)	0.01398 (9)	0.01023 (9)	0.01176 (9)
S1	0.0413 (7)	0.0448 (8)	0.0476 (8)	0.0104 (6)	−0.0010 (6)	0.0044 (6)
S2	0.0536 (9)	0.0512 (9)	0.0505 (8)	0.0173 (7)	0.0237 (7)	0.0150 (7)
P1	0.0381 (7)	0.0436 (8)	0.0396 (7)	0.0055 (6)	0.0030 (6)	0.0122 (6)
P2	0.0404 (7)	0.0411 (8)	0.0356 (7)	0.0125 (6)	0.0090 (6)	0.0098 (6)
O1	0.048 (2)	0.050 (2)	0.043 (2)	0.0185 (17)	−0.0063 (17)	−0.0009 (17)
O2	0.075 (3)	0.054 (3)	0.058 (3)	0.021 (2)	0.024 (2)	0.022 (2)
N1	0.048 (3)	0.040 (3)	0.040 (2)	0.008 (2)	0.003 (2)	0.004 (2)
N2	0.066 (3)	0.055 (3)	0.050 (3)	0.012 (3)	0.016 (3)	0.007 (2)
C1	0.042 (3)	0.044 (3)	0.038 (3)	0.009 (2)	0.005 (2)	0.014 (2)
C2	0.049 (3)	0.047 (3)	0.051 (3)	0.006 (3)	−0.007 (3)	0.006 (3)
C3	0.062 (5)	0.105 (7)	0.116 (7)	−0.016 (4)	−0.005 (5)	0.040 (5)
C4	0.107 (6)	0.084 (6)	0.052 (4)	0.018 (5)	−0.015 (4)	−0.002 (4)
C5	0.048 (3)	0.054 (4)	0.061 (4)	0.020 (3)	−0.001 (3)	0.004 (3)
C6	0.038 (3)	0.048 (3)	0.040 (3)	0.003 (2)	−0.001 (2)	0.015 (2)
C7	0.038 (3)	0.053 (3)	0.049 (3)	0.007 (2)	0.000 (2)	0.022 (3)
C8	0.037 (3)	0.077 (5)	0.061 (4)	0.005 (3)	0.000 (3)	0.030 (3)
C9	0.037 (3)	0.082 (5)	0.084 (5)	0.006 (3)	0.008 (3)	0.050 (4)
C10	0.045 (4)	0.072 (5)	0.105 (6)	0.025 (3)	0.011 (3)	0.051 (4)
C11	0.032 (3)	0.061 (4)	0.071 (4)	0.010 (3)	0.009 (3)	0.033 (3)
C12	0.059 (5)	0.115 (7)	0.165 (9)	0.015 (4)	0.021 (5)	0.100 (7)
C13	0.045 (3)	0.042 (3)	0.036 (3)	0.007 (2)	0.002 (2)	0.010 (2)
C14	0.051 (3)	0.053 (4)	0.045 (3)	0.002 (3)	0.000 (3)	0.012 (3)
C15	0.072 (4)	0.055 (4)	0.039 (3)	0.002 (3)	−0.002 (3)	0.002 (3)
C16	0.079 (5)	0.051 (4)	0.043 (3)	0.012 (3)	0.016 (3)	0.014 (3)
C17	0.083 (5)	0.059 (4)	0.058 (4)	−0.003 (4)	0.025 (4)	0.019 (3)
C18	0.066 (4)	0.047 (4)	0.046 (3)	0.000 (3)	0.013 (3)	0.008 (3)
C19	0.115 (7)	0.093 (6)	0.054 (4)	0.002 (5)	0.027 (4)	0.005 (4)
C20	0.039 (3)	0.048 (3)	0.045 (3)	0.005 (2)	0.003 (2)	0.018 (3)
C21	0.044 (3)	0.058 (4)	0.058 (4)	0.010 (3)	−0.001 (3)	0.011 (3)
C22	0.059 (4)	0.057 (4)	0.071 (4)	0.018 (3)	0.008 (3)	0.010 (3)
C23	0.038 (3)	0.072 (4)	0.065 (4)	0.013 (3)	0.014 (3)	0.037 (3)
C24	0.050 (4)	0.080 (5)	0.072 (4)	0.014 (3)	−0.007 (3)	0.029 (4)
C25	0.060 (4)	0.062 (4)	0.050 (4)	0.012 (3)	−0.008 (3)	0.010 (3)
C26	0.051 (4)	0.099 (6)	0.094 (6)	0.029 (4)	0.009 (4)	0.040 (5)
C27	0.032 (3)	0.056 (4)	0.058 (4)	0.009 (2)	0.005 (3)	0.020 (3)
C28	0.131 (7)	0.050 (4)	0.069 (5)	0.016 (4)	0.044 (5)	0.003 (3)
C29	0.127 (9)	0.238 (14)	0.109 (8)	0.105 (9)	0.034 (7)	0.089 (9)
C30	0.093 (6)	0.161 (9)	0.060 (5)	−0.005 (6)	0.028 (4)	−0.001 (5)
C31	0.083 (5)	0.056 (4)	0.085 (5)	0.015 (4)	0.029 (4)	0.030 (4)
C32	0.037 (3)	0.043 (3)	0.041 (3)	0.013 (2)	0.007 (2)	0.012 (2)
C33	0.051 (3)	0.049 (3)	0.043 (3)	0.014 (3)	0.010 (3)	0.010 (3)
C34	0.057 (4)	0.058 (4)	0.040 (3)	0.024 (3)	0.008 (3)	0.012 (3)
C35	0.049 (3)	0.058 (4)	0.043 (3)	0.028 (3)	0.002 (3)	0.006 (3)
C36	0.052 (4)	0.050 (4)	0.073 (4)	−0.002 (3)	−0.004 (3)	0.017 (3)
C37	0.055 (4)	0.060 (4)	0.051 (3)	0.013 (3)	0.007 (3)	0.026 (3)
C38	0.069 (4)	0.070 (5)	0.065 (4)	0.019 (3)	−0.014 (3)	−0.007 (3)
C39	0.042 (3)	0.049 (3)	0.035 (3)	0.014 (2)	0.013 (2)	0.016 (2)
C40	0.047 (3)	0.049 (4)	0.056 (4)	0.011 (3)	0.006 (3)	0.011 (3)
C41	0.053 (4)	0.044 (4)	0.079 (4)	0.017 (3)	0.018 (3)	0.017 (3)
C42	0.045 (3)	0.073 (4)	0.066 (4)	0.029 (3)	0.026 (3)	0.039 (3)
C43	0.047 (3)	0.080 (5)	0.051 (4)	0.014 (3)	0.007 (3)	0.027 (3)
C44	0.052 (3)	0.056 (4)	0.044 (3)	0.020 (3)	0.009 (3)	0.011 (3)
C45	0.059 (4)	0.096 (6)	0.095 (5)	0.045 (4)	0.027 (4)	0.039 (5)
C46	0.042 (3)	0.042 (3)	0.034 (3)	0.012 (2)	0.008 (2)	0.008 (2)
C47	0.056 (4)	0.047 (3)	0.052 (3)	0.019 (3)	0.019 (3)	0.008 (3)
C48	0.053 (4)	0.054 (4)	0.053 (3)	0.012 (3)	0.022 (3)	0.016 (3)
C49	0.049 (3)	0.042 (3)	0.044 (3)	0.004 (2)	0.008 (3)	0.009 (2)
C50	0.052 (3)	0.038 (3)	0.051 (3)	0.012 (2)	0.003 (3)	0.008 (2)
C51	0.041 (3)	0.048 (3)	0.043 (3)	0.012 (2)	0.007 (2)	0.006 (2)
C52	0.083 (5)	0.051 (4)	0.066 (4)	0.006 (3)	0.024 (4)	0.019 (3)

### Geometric parameters (Å, °)

**Table:**

Au1—S1	2.3221 (13)	C22—C23	1.366 (8)
Au1—P1	2.2638 (13)	C22—H22	0.9400
Au1—Au2	3.1351 (3)	C23—C24	1.391 (9)
Au2—S2	2.3102 (14)	C23—C26	1.496 (8)
Au2—P2	2.2589 (14)	C24—C25	1.374 (8)
S1—C1	1.763 (6)	C24—H24	0.9400
S2—C27	1.768 (6)	C25—H25	0.9400
P1—C13	1.801 (5)	C26—H26A	0.9700
P1—C6	1.816 (5)	C26—H26B	0.9700
P1—C20	1.818 (5)	C26—H26C	0.9700
P2—C32	1.811 (5)	C28—C29	1.467 (11)
P2—C46	1.812 (5)	C28—C30	1.511 (11)
P2—C39	1.813 (5)	C28—H28	0.9900
O1—C1	1.370 (6)	C29—H29A	0.9700
O1—C5	1.427 (6)	C29—H29B	0.9700
O2—C27	1.377 (6)	C29—H29C	0.9700
O2—C31	1.425 (7)	C30—H30A	0.9700
N1—C1	1.261 (6)	C30—H30B	0.9700
N1—C2	1.462 (6)	C30—H30C	0.9700
N2—C27	1.255 (7)	C31—H31A	0.9700
N2—C28	1.449 (8)	C31—H31B	0.9700
C2—C3	1.487 (8)	C31—H31C	0.9700
C2—C4	1.502 (8)	C32—C37	1.387 (7)
C2—H2	0.9900	C32—C33	1.389 (7)
C3—H3A	0.9700	C33—C34	1.368 (7)
C3—H3B	0.9700	C33—H33	0.9400
C3—H3C	0.9700	C34—C35	1.383 (8)
C4—H4A	0.9700	C34—H34	0.9400
C4—H4B	0.9700	C35—C36	1.374 (8)
C4—H4C	0.9700	C35—C38	1.502 (8)
C5—H5A	0.9700	C36—C37	1.368 (8)
C5—H5B	0.9700	C36—H36	0.9400
C5—H5C	0.9700	C37—H37	0.9400
C6—C7	1.377 (7)	C38—H38A	0.9700
C6—C11	1.390 (7)	C38—H38B	0.9700
C7—C8	1.389 (7)	C38—H38C	0.9700
C7—H7	0.9400	C39—C44	1.387 (7)
C8—C9	1.367 (8)	C39—C40	1.388 (8)
C8—H8	0.9400	C40—C41	1.378 (8)
C9—C10	1.384 (8)	C40—H40	0.9400
C9—C12	1.506 (8)	C41—C42	1.385 (8)
C10—C11	1.367 (8)	C41—H41	0.9400
C10—H10	0.9400	C42—C43	1.376 (9)
C11—H11	0.9400	C42—C45	1.519 (8)
C12—H12A	0.9700	C43—C44	1.373 (8)
C12—H12B	0.9700	C43—H43	0.9400
C12—H12C	0.9700	C44—H44	0.9400
C13—C18	1.383 (7)	C45—H45A	0.9700
C13—C14	1.386 (7)	C45—H45B	0.9700
C14—C15	1.377 (8)	C45—H45C	0.9700
C14—H14	0.9400	C46—C51	1.383 (7)
C15—C16	1.378 (8)	C46—C47	1.390 (7)
C15—H15	0.9400	C47—C48	1.385 (7)
C16—C17	1.383 (8)	C47—H47	0.9400
C16—C19	1.507 (8)	C48—C49	1.380 (8)
C17—C18	1.387 (8)	C48—H48	0.9400
C17—H17	0.9400	C49—C50	1.380 (7)
C18—H18	0.9400	C49—C52	1.503 (7)
C19—H19A	0.9700	C50—C51	1.386 (7)
C19—H19B	0.9700	C50—H50	0.9400
C19—H19C	0.9700	C51—H51	0.9400
C20—C21	1.379 (8)	C52—H52A	0.9700
C20—C25	1.390 (7)	C52—H52B	0.9700
C21—C22	1.387 (8)	C52—H52C	0.9700
C21—H21	0.9400		
			
P1—Au1—S1	175.31 (5)	C25—C24—C23	122.2 (6)
P1—Au1—Au2	106.83 (4)	C25—C24—H24	118.9
S1—Au1—Au2	77.57 (3)	C23—C24—H24	118.9
P2—Au2—S2	176.45 (5)	C24—C25—C20	120.6 (6)
P2—Au2—Au1	100.80 (3)	C24—C25—H25	119.7
S2—Au2—Au1	81.87 (4)	C20—C25—H25	119.7
C1—S1—Au1	102.97 (17)	C23—C26—H26A	109.5
C27—S2—Au2	102.9 (2)	C23—C26—H26B	109.5
C13—P1—C6	105.5 (3)	H26A—C26—H26B	109.5
C13—P1—C20	106.0 (2)	C23—C26—H26C	109.5
C6—P1—C20	105.8 (2)	H26A—C26—H26C	109.5
C13—P1—Au1	114.69 (18)	H26B—C26—H26C	109.5
C6—P1—Au1	113.50 (16)	N2—C27—O2	118.8 (5)
C20—P1—Au1	110.72 (18)	N2—C27—S2	129.0 (5)
C32—P2—C46	105.8 (2)	O2—C27—S2	112.2 (4)
C32—P2—C39	104.9 (2)	N2—C28—C29	109.2 (7)
C46—P2—C39	105.3 (2)	N2—C28—C30	109.0 (7)
C32—P2—Au2	114.84 (18)	C29—C28—C30	108.9 (7)
C46—P2—Au2	111.00 (17)	N2—C28—H28	109.9
C39—P2—Au2	114.20 (16)	C29—C28—H28	109.9
C1—O1—C5	116.3 (4)	C30—C28—H28	109.9
C27—O2—C31	116.3 (5)	C28—C29—H29A	109.5
C1—N1—C2	118.7 (5)	C28—C29—H29B	109.5
C27—N2—C28	119.5 (6)	H29A—C29—H29B	109.5
N1—C1—O1	119.8 (5)	C28—C29—H29C	109.5
N1—C1—S1	128.8 (4)	H29A—C29—H29C	109.5
O1—C1—S1	111.4 (4)	H29B—C29—H29C	109.5
N1—C2—C3	109.4 (5)	C28—C30—H30A	109.5
N1—C2—C4	108.6 (5)	C28—C30—H30B	109.5
C3—C2—C4	112.1 (6)	H30A—C30—H30B	109.5
N1—C2—H2	108.9	C28—C30—H30C	109.5
C3—C2—H2	108.9	H30A—C30—H30C	109.5
C4—C2—H2	108.9	H30B—C30—H30C	109.5
C2—C3—H3A	109.5	O2—C31—H31A	109.5
C2—C3—H3B	109.5	O2—C31—H31B	109.5
H3A—C3—H3B	109.5	H31A—C31—H31B	109.5
C2—C3—H3C	109.5	O2—C31—H31C	109.5
H3A—C3—H3C	109.5	H31A—C31—H31C	109.5
H3B—C3—H3C	109.5	H31B—C31—H31C	109.5
C2—C4—H4A	109.5	C37—C32—C33	117.2 (5)
C2—C4—H4B	109.5	C37—C32—P2	119.3 (4)
H4A—C4—H4B	109.5	C33—C32—P2	123.4 (4)
C2—C4—H4C	109.5	C34—C33—C32	120.7 (5)
H4A—C4—H4C	109.5	C34—C33—H33	119.6
H4B—C4—H4C	109.5	C32—C33—H33	119.6
O1—C5—H5A	109.5	C33—C34—C35	122.0 (5)
O1—C5—H5B	109.5	C33—C34—H34	119.0
H5A—C5—H5B	109.5	C35—C34—H34	119.0
O1—C5—H5C	109.5	C36—C35—C34	117.0 (5)
H5A—C5—H5C	109.5	C36—C35—C38	122.2 (6)
H5B—C5—H5C	109.5	C34—C35—C38	120.8 (6)
C7—C6—C11	119.0 (5)	C37—C36—C35	121.8 (6)
C7—C6—P1	122.1 (4)	C37—C36—H36	119.1
C11—C6—P1	118.4 (4)	C35—C36—H36	119.1
C6—C7—C8	120.3 (5)	C36—C37—C32	121.2 (5)
C6—C7—H7	119.9	C36—C37—H37	119.4
C8—C7—H7	119.9	C32—C37—H37	119.4
C9—C8—C7	120.9 (6)	C35—C38—H38A	109.5
C9—C8—H8	119.6	C35—C38—H38B	109.5
C7—C8—H8	119.6	H38A—C38—H38B	109.5
C8—C9—C10	118.3 (6)	C35—C38—H38C	109.5
C8—C9—C12	120.2 (6)	H38A—C38—H38C	109.5
C10—C9—C12	121.4 (6)	H38B—C38—H38C	109.5
C11—C10—C9	121.7 (6)	C44—C39—C40	118.0 (5)
C11—C10—H10	119.1	C44—C39—P2	118.5 (4)
C9—C10—H10	119.1	C40—C39—P2	123.5 (4)
C10—C11—C6	119.8 (5)	C41—C40—C39	120.5 (5)
C10—C11—H11	120.1	C41—C40—H40	119.8
C6—C11—H11	120.1	C39—C40—H40	119.8
C9—C12—H12A	109.5	C40—C41—C42	121.5 (6)
C9—C12—H12B	109.5	C40—C41—H41	119.2
H12A—C12—H12B	109.5	C42—C41—H41	119.2
C9—C12—H12C	109.5	C43—C42—C41	117.5 (5)
H12A—C12—H12C	109.5	C43—C42—C45	122.2 (6)
H12B—C12—H12C	109.5	C41—C42—C45	120.3 (6)
C18—C13—C14	117.9 (5)	C44—C43—C42	121.7 (6)
C18—C13—P1	118.2 (4)	C44—C43—H43	119.1
C14—C13—P1	123.7 (4)	C42—C43—H43	119.1
C15—C14—C13	121.1 (6)	C43—C44—C39	120.8 (6)
C15—C14—H14	119.4	C43—C44—H44	119.6
C13—C14—H14	119.4	C39—C44—H44	119.6
C14—C15—C16	121.1 (5)	C42—C45—H45A	109.5
C14—C15—H15	119.5	C42—C45—H45B	109.5
C16—C15—H15	119.5	H45A—C45—H45B	109.5
C15—C16—C17	118.1 (6)	C42—C45—H45C	109.5
C15—C16—C19	121.3 (6)	H45A—C45—H45C	109.5
C17—C16—C19	120.5 (6)	H45B—C45—H45C	109.5
C16—C17—C18	121.0 (6)	C51—C46—C47	118.6 (5)
C16—C17—H17	119.5	C51—C46—P2	119.2 (4)
C18—C17—H17	119.5	C47—C46—P2	122.1 (4)
C13—C18—C17	120.7 (5)	C48—C47—C46	120.5 (5)
C13—C18—H18	119.6	C48—C47—H47	119.7
C17—C18—H18	119.6	C46—C47—H47	119.7
C16—C19—H19A	109.5	C49—C48—C47	121.2 (6)
C16—C19—H19B	109.5	C49—C48—H48	119.4
H19A—C19—H19B	109.5	C47—C48—H48	119.4
C16—C19—H19C	109.5	C48—C49—C50	117.8 (5)
H19A—C19—H19C	109.5	C48—C49—C52	120.5 (6)
H19B—C19—H19C	109.5	C50—C49—C52	121.6 (5)
C21—C20—C25	117.8 (5)	C49—C50—C51	121.8 (5)
C21—C20—P1	119.4 (4)	C49—C50—H50	119.1
C25—C20—P1	122.8 (5)	C51—C50—H50	119.1
C20—C21—C22	120.3 (5)	C46—C51—C50	120.0 (5)
C20—C21—H21	119.8	C46—C51—H51	120.0
C22—C21—H21	119.8	C50—C51—H51	120.0
C23—C22—C21	122.7 (6)	C49—C52—H52A	109.5
C23—C22—H22	118.7	C49—C52—H52B	109.5
C21—C22—H22	118.7	H52A—C52—H52B	109.5
C22—C23—C24	116.3 (6)	C49—C52—H52C	109.5
C22—C23—C26	122.2 (7)	H52A—C52—H52C	109.5
C24—C23—C26	121.5 (6)	H52B—C52—H52C	109.5
			
P1—Au1—Au2—P2	−98.27 (5)	C21—C22—C23—C26	−179.7 (6)
S1—Au1—Au2—P2	80.05 (5)	C22—C23—C24—C25	−1.4 (10)
P1—Au1—Au2—S2	79.36 (6)	C26—C23—C24—C25	179.7 (6)
S1—Au1—Au2—S2	−102.32 (5)	C23—C24—C25—C20	0.2 (10)
Au2—Au1—S1—C1	−137.47 (18)	C21—C20—C25—C24	1.1 (9)
Au1—Au2—S2—C27	−130.07 (18)	P1—C20—C25—C24	179.1 (5)
Au2—Au1—P1—C13	−94.31 (19)	C28—N2—C27—O2	179.8 (6)
Au2—Au1—P1—C6	27.1 (2)	C28—N2—C27—S2	−1.2 (9)
Au2—Au1—P1—C20	145.85 (18)	C31—O2—C27—N2	−1.2 (8)
Au1—Au2—P2—C32	−114.68 (19)	C31—O2—C27—S2	179.6 (4)
Au1—Au2—P2—C46	125.42 (17)	Au2—S2—C27—N2	160.8 (5)
Au1—Au2—P2—C39	6.5 (2)	Au2—S2—C27—O2	−20.1 (4)
C2—N1—C1—O1	177.7 (5)	C27—N2—C28—C29	−110.8 (8)
C2—N1—C1—S1	−2.2 (8)	C27—N2—C28—C30	130.4 (6)
C5—O1—C1—N1	0.8 (7)	C46—P2—C32—C37	169.9 (5)
C5—O1—C1—S1	−179.2 (4)	C39—P2—C32—C37	−79.1 (5)
Au1—S1—C1—N1	168.1 (5)	Au2—P2—C32—C37	47.1 (5)
Au1—S1—C1—O1	−11.9 (4)	C46—P2—C32—C33	−12.9 (5)
C1—N1—C2—C3	98.3 (7)	C39—P2—C32—C33	98.2 (5)
C1—N1—C2—C4	−139.0 (6)	Au2—P2—C32—C33	−135.7 (4)
C13—P1—C6—C7	−126.4 (4)	C37—C32—C33—C34	1.1 (8)
C20—P1—C6—C7	−14.4 (5)	P2—C32—C33—C34	−176.1 (4)
Au1—P1—C6—C7	107.2 (4)	C32—C33—C34—C35	1.4 (9)
C13—P1—C6—C11	61.8 (5)	C33—C34—C35—C36	−3.0 (9)
C20—P1—C6—C11	173.8 (5)	C33—C34—C35—C38	176.3 (5)
Au1—P1—C6—C11	−64.6 (5)	C34—C35—C36—C37	2.2 (9)
C11—C6—C7—C8	−1.0 (8)	C38—C35—C36—C37	−177.1 (6)
P1—C6—C7—C8	−172.7 (4)	C35—C36—C37—C32	0.3 (10)
C6—C7—C8—C9	1.1 (9)	C33—C32—C37—C36	−2.0 (9)
C7—C8—C9—C10	−1.2 (10)	P2—C32—C37—C36	175.4 (5)
C7—C8—C9—C12	178.0 (6)	C32—P2—C39—C44	−170.2 (4)
C8—C9—C10—C11	1.2 (11)	C46—P2—C39—C44	−58.8 (5)
C12—C9—C10—C11	−177.9 (7)	Au2—P2—C39—C44	63.2 (5)
C9—C10—C11—C6	−1.1 (10)	C32—P2—C39—C40	11.2 (5)
C7—C6—C11—C10	1.0 (9)	C46—P2—C39—C40	122.6 (5)
P1—C6—C11—C10	173.0 (5)	Au2—P2—C39—C40	−115.4 (4)
C6—P1—C13—C18	−149.1 (4)	C44—C39—C40—C41	−2.0 (8)
C20—P1—C13—C18	99.0 (5)	P2—C39—C40—C41	176.6 (5)
Au1—P1—C13—C18	−23.4 (5)	C39—C40—C41—C42	1.3 (9)
C6—P1—C13—C14	34.5 (5)	C40—C41—C42—C43	−0.2 (9)
C20—P1—C13—C14	−77.4 (5)	C40—C41—C42—C45	179.2 (6)
Au1—P1—C13—C14	160.2 (4)	C41—C42—C43—C44	0.0 (9)
C18—C13—C14—C15	−1.9 (9)	C45—C42—C43—C44	−179.4 (6)
P1—C13—C14—C15	174.5 (5)	C42—C43—C44—C39	−0.8 (9)
C13—C14—C15—C16	0.8 (10)	C40—C39—C44—C43	1.8 (8)
C14—C15—C16—C17	1.5 (10)	P2—C39—C44—C43	−176.9 (4)
C14—C15—C16—C19	178.6 (6)	C32—P2—C46—C51	−98.7 (4)
C15—C16—C17—C18	−2.8 (10)	C39—P2—C46—C51	150.6 (4)
C19—C16—C17—C18	−179.8 (7)	Au2—P2—C46—C51	26.5 (4)
C14—C13—C18—C17	0.7 (9)	C32—P2—C46—C47	78.6 (5)
P1—C13—C18—C17	−175.9 (5)	C39—P2—C46—C47	−32.1 (5)
C16—C17—C18—C13	1.6 (11)	Au2—P2—C46—C47	−156.2 (4)
C13—P1—C20—C21	−142.6 (5)	C51—C46—C47—C48	−1.0 (8)
C6—P1—C20—C21	105.7 (5)	P2—C46—C47—C48	−178.3 (4)
Au1—P1—C20—C21	−17.6 (5)	C46—C47—C48—C49	2.7 (9)
C13—P1—C20—C25	39.5 (5)	C47—C48—C49—C50	−2.3 (8)
C6—P1—C20—C25	−72.2 (5)	C47—C48—C49—C52	−179.5 (5)
Au1—P1—C20—C25	164.4 (4)	C48—C49—C50—C51	0.3 (8)
C25—C20—C21—C22	−1.1 (9)	C52—C49—C50—C51	177.5 (5)
P1—C20—C21—C22	−179.2 (5)	C47—C46—C51—C50	−1.0 (7)
C20—C21—C22—C23	−0.2 (10)	P2—C46—C51—C50	176.4 (4)
C21—C22—C23—C24	1.4 (10)	C49—C50—C51—C46	1.3 (8)
